# Site-Specific Patterns of Distant Relapse on ^18F-FDG PET/Contrast-Enhanced CT According to Molecular Subtype in Breast Cancer: A Retrospective Cohort Study

**DOI:** 10.1007/s13139-026-01022-7

**Published:** 2026-05-07

**Authors:** Agostino Chiaravalloti, Gianluca Vanni, Luca Verdesca, Daniele Di Biagio, Mario Tavolozza, Oreste Claudio Buonomo, Orazio Schillaci

**Affiliations:** 1 Department of Biomedicine and Prevention, University of Rome Tor Vergata, Via Montpellier 1, Rome, 00133 Italy; 2 IRCCS Neuromed, Via Atinense, 18 - 86077, Pozzilli, Italy; 3 Department of Surgical Sciences, University of Rome Tor Vergata, Rome, Italy; 4 Ospedale San Pietro Fatebenefratelli, Rome, Italy; 5 UOC Medicina Nucleare, Policlinico Tor Vergata, Rome, Italy; 6 Università degli Studi della Basilicata, Potenza, Italy

**Keywords:** Breast cancer, ^18F-FDG PET/CT, Recurrence, Distant metastases, Molecular subtype, Restaging

## Abstract

**Purpose:**

To describe the site-specific distribution of distant relapse detected by integrated ^18F-FDG PET/contrast-enhanced CT (PET/ceCT) in breast cancer patients and to explore its association with molecular subtype and clinicopathological features.

**Methods:**

This retrospective study included 177 postoperative breast cancer patients who underwent PET/ceCT during follow-up/restaging for suspected recurrence. Clinical and pathological data were extracted from a manually curated institutional database, including type of surgery, pathological T stage, pathological nodal status, histology, molecular subtype, PET/ceCT date, and PET/ceCT-detected site of relapse. Site-specific analyses were performed in patients with codable distant relapse.

**Results:**

A PET/ceCT relapse-site entry was available in 141/177 patients (79.7%), and 137/177 (77.4%) had codable distant relapse. Molecular subtype was available in 166/177 patients (93.8%). Bone was the most frequent site of distant relapse (81/137, 59.1%), followed by lung (44/137, 32.1%), distant lymph nodes (41/137, 29.9%), and liver (40/137, 29.2%); brain involvement was uncommon (8/137, 5.8%). Single-site and multisite relapse were observed in 70/137 (51.1%) and 67/137 (48.9%) patients, respectively. Bone involvement was significantly more frequent in luminal than in non-luminal tumors (65/101, 64.4% vs 11/29, 37.9%; p=0.018).

**Conclusion:**

PET/ceCT disclosed non-random and biologically meaningful patterns of distant relapse in breast cancer. Bone-dominant relapse was the prevailing phenotype overall and was significantly associated with luminal disease, whereas non-luminal tumors showed relatively more visceral and multisite dissemination.

## Introduction

Breast cancer is a biologically heterogeneous disease, and recurrence assessment remains clinically relevant because the extent and distribution of relapse may directly affect systemic treatment selection, local treatment options, and overall therapeutic strategy. In current practice, imaging during follow-up is mainly performed when recurrence is clinically suspected, when conventional imaging is equivocal, or when tumour markers increase. In this setting, whole-body hybrid imaging is particularly attractive because it can simultaneously assess locoregional disease and distant spread [1]. Among available techniques, ^18F-FDG PET/CT has shown good diagnostic performance for the detection of breast cancer recurrence. In a review of the available evidence, the pooled sensitivity of ^18F-FDG PET in an early meta-analysis was 90%, while a later meta-analysis including 26 studies and 1,752 patients with suspected recurrence reported pooled sensitivity and specificity of 0.90 and 0.81, respectively. In addition, in a prospective comparison cited in the same review, the area under the ROC curve for distant recurrence was 0.99 for ^18F-FDG PET/CT, compared with 0.84 for contrast-enhanced CT and 0.86 for the combination of contrast-enhanced CT and bone scintigraphy [1].

The clinical relevance of early whole-body assessment is further supported by studies performed in selected follow-up populations. In asymptomatic breast cancer patients with a reproducible tumour marker increase, whole-body imaging identified metastatic disease in 29 of 44 cases (65.9%), while limited metastatic burden was found in 7 of 29 patients (24.1%). In that cohort, bone was the most frequent metastatic site, accounting for 62.1% of metastatic cases [2]. These findings suggest that recurrence imaging should not be limited to confirming disease presence, but should also define the anatomical distribution of relapse, as this may influence subsequent management [2]. At the same time, FDG uptake is not independent of tumour biology. Previous studies have shown significant associations between glucose metabolism and histological grade, histological type, tumour size, and hormone receptor status. More broadly, ^18F-FDG PET/CT is known to perform less well in low-proliferative, low-grade, and well-differentiated luminal tumours, whereas more aggressive subtypes generally show higher metabolic activity [1, 3]. Thus, imaging findings in recurrent breast cancer are likely to reflect not only tumour burden, but also underlying biological heterogeneity [1, 3]. This concept is also relevant in the recurrent setting. In a bicentric series of 179 patients with recurrent luminal breast cancer, ^18F-FDG PET/CT was suggestive of recurrence in 114 cases (63.7%) and was associated with significantly shorter overall survival, independently of CA 15.3 levels [4]. Therefore, PET/CT may capture clinically meaningful relapse phenotypes even within biologically less FDG-avid disease [4]. However, the existing literature has mainly focused on diagnostic accuracy, prognostic stratification, or comparison with conventional imaging, whereas less attention has been paid to whether established biological patterns of site-specific metastasis across breast cancer subtypes are consistently reflected in findings from integrated ^18F-FDG PET/contrast-enhanced CT (PET/ceCT) in real-world practice. Therefore, the aim of the present study was to describe the site-specific distribution of distant relapse detected by PET/ceCT and to assess its relationship with known subtype-related metastatic patterns [1, 2, 4].

## Materials and Methods

### Study Design and Patient Population

This retrospective observational study included postoperative breast cancer patients who underwent PET/ceCT during follow-up/restaging for suspected recurrence at our institution. Clinical and pathological data were retrieved from an institutional database specifically curated for the present analysis. Only patients with histologically proven breast cancer and available PET/ceCT-based relapse information were considered eligible. Consistent with previously published PET/CT series in recurrent breast cancer, patients were included if PET/CT had been performed in the postoperative setting for the assessment of suspected recurrent disease [5, 6].

For the current study, the dataset was manually revised and harmonized according to predefined variables. Based on the curated database used for manuscript preparation, the following variables were extracted: type of primary surgery, pathological tumour stage (pT), pathological nodal status (pN), histological subtype, molecular subtype, date of PET/ceCT, and PET/ceCT-detected site of relapse. Cases with incomplete clinicopathological annotation were retained for descriptive analyses whenever possible, whereas patients with non-codable or ambiguous relapse-site entries were excluded from organ-specific distant-relapse analyses.

## Clinicopathological Data

Type of primary surgery was classified into breast-conserving surgery, mastectomy, biopsy-only/diagnostic procedure, or other surgery according to the original database entry. Pathological tumour stage and nodal status were recorded as reported in the surgical pathology records. Histology was harmonized into the following categories: ductal carcinoma, lobular carcinoma, mixed ductal-lobular carcinoma, mucinous carcinoma, clear-cell carcinoma, poorly differentiated/indeterminate carcinoma, and other less frequent histologies.

Molecular subtype was categorized according to the available biomolecular classification in the database into Luminal A, Luminal B/HER2-negative, Luminal B/HER2-positive, HER2-enriched, and basal-like/triple-negative disease. This approach is consistent with subtype-based stratification commonly used in breast cancer PET studies and in prior clinicopathological ^18F-FDG PET/C analyses [6].

## Definition of PET/ceCT-Detected Relapse Pattern

The database field describing the site of metastatic disease was considered the PET/ceCT-detected site of recurrence. Original free-text entries were manually reviewed and recoded into predefined distant-relapse categories. The main organ/system categories were bone, lung, liver, brain, and distant lymph nodes. Less frequent sites, when clearly identifiable, were grouped into other distant sites. Cases limited to locoregional recurrence or entries not allowing a reliable classification as distant disease were not included in the site-specific distant-relapse analysis.

Patients with one single distant site category were classified as having single-site relapse, whereas those with more than one distinct distant category were classified as having multisite relapse. The date reported in the database for the PET examination was used as the imaging reference date for relapse assessment [6].

## PET/CT Acquisition Protocol

^18F-FDG PET/CT acquisition followed the same institutional protocol previously described in our published experience. Patients fasted for at least 5 h before intravenous ^18F-FDG administration. Serum glucose levels were checked before tracer injection. Patients received 370–450 MBq of ^18F-FDG intravenously and were hydrated with 500 mL of intravenous saline. Physical activity was kept to a minimum, and image acquisition started approximately 60 min after tracer injection. PET/CT examinations were performed on a Discovery IQ system (GE Medical Systems, Fairfield, CT, USA). A low-dose CT scan was first acquired for attenuation correction, followed by whole-body PET acquisition in the caudocranial direction from the upper thighs to the vertex, with an acquisition time of 3.5 min per bed position. PET data were reconstructed using the Q.Clear algorithm [6].

## Contrast-Enhanced CT Component

In the present study, the diagnostic contrast-enhanced CT was performed on the same PET/CT system during the same examination session and was interpreted together with PET as a single integrated PET/ceCT examination, rather than as a separate Radiology scan. According to our institutional protocol, ceCT was acquired after intravenous administration of a nonionic iodinated contrast agent (100–120 mL, 370 mgI/mL; 420 mgI/kg) at a rate of 3 mL/s. Post-contrast CT acquisition was obtained in successive phases, with scans acquired after 30 s and 60 s from the start of contrast injection. The contrast-enhanced CT component was used together with PET for anatomical localization and lesion characterization. This distinction is important because the present work evaluates the relapse pattern detected by PET/ceCT, rather than the isolated performance of the PET component alone. This protocol is consistent with our previously published institutional experience on PET/ceCT performed in the same examination session [5, 6].

### Image Interpretation

PET/ceCT images were reviewed on a dedicated workstation allowing separate and fused visualization of PET and CT datasets in axial, coronal, and sagittal planes. Image interpretation reflected routine clinical practice rather than a predefined study-specific double-reading protocol. In routine practice, PET/ceCT findings were interpreted side by side by a nuclear medicine physician and a radiologist, both with at least 10 years of experience. Focal tracer uptake higher than background and not attributable to physiological distribution or benign processes was considered suspicious for disease involvement. No absolute SUV cut-off was used for lesion classification. In equivocal or mildly avid lesions, co-registered CT findings were used to improve lesion delineation and interpretation. Metastatic lesions were distinguished from common benign pitfalls, including fractures and degenerative changes, on the basis of the integrated PET/ceCT assessment, taking into account lesion morphology, anatomical distribution, and correlation with subsequent imaging follow-up when available. The final clinical report was based on the integrated assessment of both readers. Because this was a retrospective analysis of routine clinical reports, the initial inter-reader disagreement rate before consensus was not systematically recorded [5, 6].

## Study Endpoints

The primary endpoint of the study was the anatomical distribution of distant relapse detected by integrated PET/ceCT. Secondary endpoints were the association between relapse pattern and clinicopathological variables, including molecular subtype, pathological tumour stage, pathological nodal status, histology, and type of surgery. The study was not designed as a head-to-head comparison with other imaging modalities and did not include lesion-by-lesion sensitivity or specificity analysis.

### Statistical Analysis

Categorical variables were expressed as absolute numbers and percentages. Comparisons between categorical variables were planned using the chi-square test or Fisher’s exact test, as appropriate. Patients with missing data for a given variable were excluded only from the corresponding analysis; no imputation procedure was applied. A two-sided p value < 0.05 was considered statistically significant [7].

## Results

### Patient Population and Data Completeness

After manual curation of the institutional database, 177 postoperative breast cancer patients were included in the study. Information on type of primary surgery was available in 148/177 patients (83.6%), pathological T stage in 135/177 (76.3%), pathological nodal status in 145/177 (81.9%), molecular subtype in 166/177 (93.8%), histological subtype in 161/177 (91.0%), and PET/ceCT date in 127/177 (71.8%). A PET/ceCT relapse-site entry was present in 141/177 patients (79.7%). After exclusion of non-informative or non-codable entries and of records referring exclusively to locoregional recurrence, 137/177 patients (77.4%) were retained for the site-specific distant-relapse analysis.

### Clinicopathological Characteristics

Among the 148 patients with available surgical data, mastectomy was the most frequent primary procedure (63/148, 42.6%), followed by breast-conserving surgery (51/148, 34.5%) and biopsy/diagnostic-only procedures (32/148, 21.6%); other surgical procedures accounted for 2/148 cases (1.4%). Among the 135 patients with available pathological T stage, pT2 was the most common category (58/135, 43.0%), followed by pT1c (42/135, 31.1%), pT4 (15/135, 11.1%), pT1b (10/135, 7.4%), and pT3 (8/135, 5.9%); pT1a and pTx were each recorded in 1 case (0.7%). Among the 145 patients with available nodal data, pathological nodal positivity predominated (94/145, 64.8%), whereas 41/145 patients (28.3%) were pN0 and 10/145 (6.9%) were pNx. Molecular subtype was available in 166 patients. Luminal B/HER2-negative disease was the most represented subgroup (66/166, 39.8%), followed by Luminal A (44/166, 26.5%), Luminal B/HER2-positive (22/166, 13.3%), HER2-enriched (19/166, 11.4%), and basal-like/triple-negative disease (15/166, 9.0%). Histology was available in 161 patients and was predominantly ductal (135/161, 83.9%). Lobular histology accounted for 14/161 cases (8.7%), whereas mucinous and mixed ductal-lobular carcinomas each represented 4/161 cases (2.5%). Poorly differentiated/indeterminate carcinoma, clear-cell carcinoma, and other rare histologies were uncommon.

### Distribution of PET/ceCT-Detected Distant Relapse

**Fig. 1:**
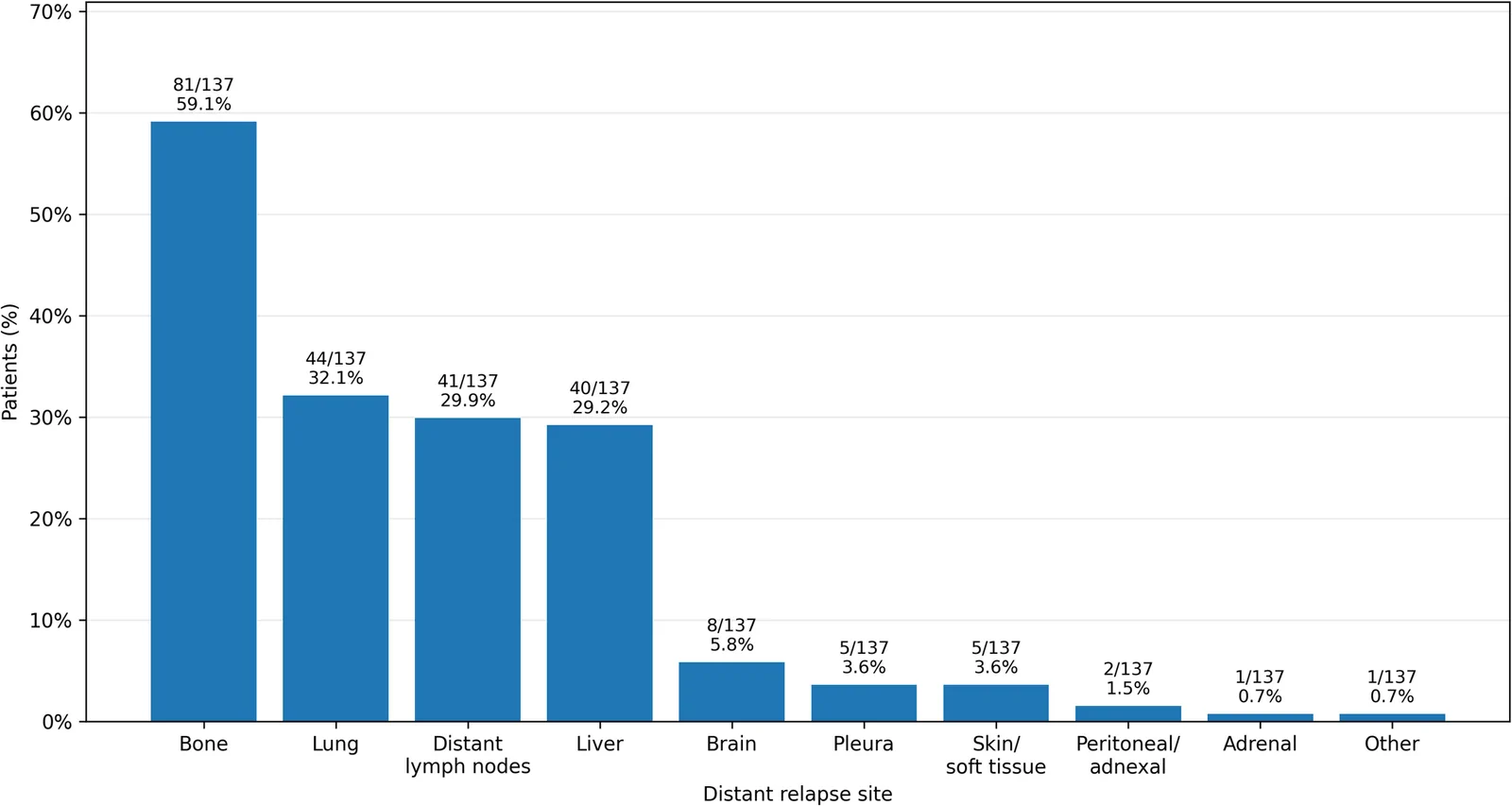
Overall distribution of distant relapse sites detected by integrated ^18F-FDG PET/contrast-enhanced CT (PET/ceCT). Among the 137 patients with codable distant relapse, bone was the most frequent site of disease involvement (59.1%), followed by lung (32.1%), distant lymph nodes (29.9%), and liver (29.2%). Brain metastases were uncommon (5.8%), while pleural, skin/soft-tissue, peritoneal/adnexal, adrenal, and other distant sites were rare. Percentages exceed 100% when summed because patients with multisite relapse contributed to more than one metastatic category

In the 137 patients with codable distant relapse on PET/ceCT, bone was the most frequent site of disease involvement, observed in 81/137 patients (59.1%). Lung involvement was detected in 44/137 cases (32.1%), distant lymph nodes in 41/137 (29.9%), and liver in 40/137 (29.2%). Brain metastases were uncommon, occurring in 8/137 patients (5.8%). Less frequent distant sites included pleural involvement in 5/137 cases (3.6%), cutaneous/soft-tissue involvement in 5/137 (3.6%), peritoneal/adnexal disease in 2/137 (1.5%), and adrenal or other distant sites in 1/137 case each (0.7%). Seventy of 137 patients (51.1%) had a single-site distant relapse, whereas 67/137 (48.9%) had multisite disease. Among single-site distant relapses, bone clearly predominated, accounting for 36/70 cases (51.4%). Single-site distant nodal relapse was observed in 12/70 patients (17.1%), followed by lung in 9/70 (12.9%) and liver in 7/70 (10.0%). Isolated brain metastases were recorded in only 2/70 patients (2.9%), while isolated pleural and isolated cutaneous/soft-tissue relapses were rare. Among patients with multisite disease, the most frequent combinations were bone plus liver (11/67, 16.4%), bone plus distant lymph nodes (8/67, 11.9%), bone plus lung (8/67, 11.9%), and distant lymph nodes plus lung (8/67, 11.9%). More complex multisite patterns involving three or four organ/system categories were less frequent but still represented a clinically relevant fraction of cases. The overall distribution of PET/ceCT-detected distant relapse sites is shown in Fig. 1.

### Relapse Pattern According to Molecular Subtype

**Fig. 2:**
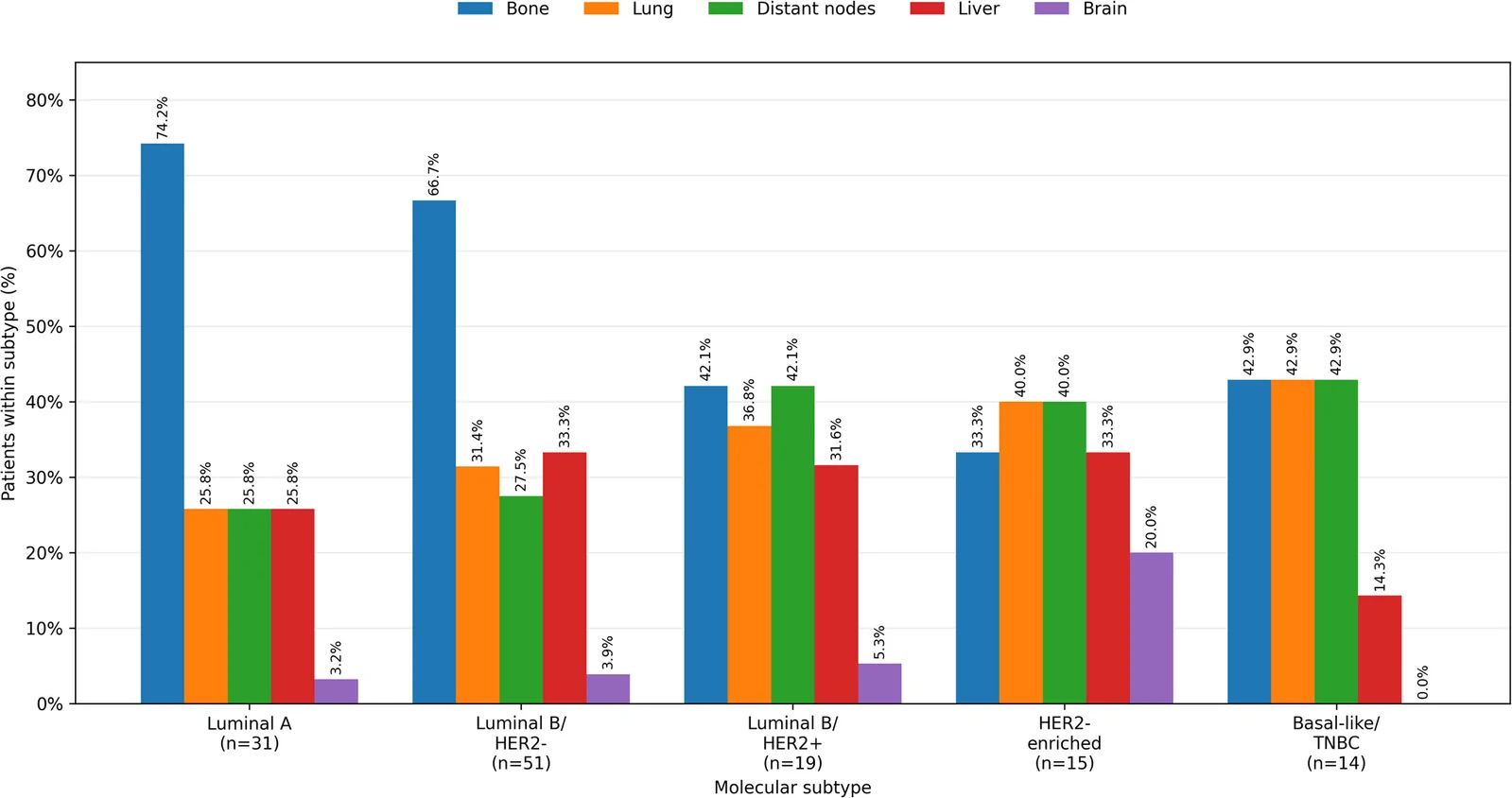
Site-specific distribution of distant relapse detected by ^18F-FDG PET/contrast-enhanced CT according to molecular subtype. Distinct subtype-related relapse patterns were observed. Bone involvement predominated in luminal tumors, particularly in Luminal A (74.2%) and Luminal B/HER2-negative disease (66.7%). By contrast, HER2-enriched and basal-like/triple-negative tumors showed relatively greater lung, distant nodal, liver, and/or brain involvement. Brain metastases were rare overall, but the highest relative frequency was observed in HER2-enriched tumors (20.0%). Percentages are calculated within each molecular subtype; categories are not mutually exclusive because patients with multisite relapse contributed to more than one metastatic **site**

Among the 130 patients with both codable distant relapse and available molecular subtype, luminal tumours showed a predominantly bone-oriented pattern. Bone involvement was observed in 65/101 luminal cases (64.4%) compared with 11/29 non-luminal cases (37.9%), and this was the only site-specific difference reaching statistical significance (*p* = 0.018). At subtype level, bone was the dominant site in Luminal A disease (23/31, 74.2%) and in Luminal B/HER2-negative disease (34/51, 66.7%). In contrast, bone involvement was less frequent in Luminal B/HER2-positive (8/19, 42.1%), HER2-enriched (5/15, 33.3%), and basal-like/triple-negative tumours (6/14, 42.9%). Visceral and nodal disease appeared relatively more common in non-luminal subtypes. Lung involvement was present in 6/15 HER2-enriched cases (40.0%) and in 6/14 basal-like/triple-negative cases (42.9%). Distant nodal relapse was also frequent in HER2-enriched (6/15, 40.0%), Luminal B/HER2-positive (8/19, 42.1%), and basal-like/triple-negative disease (6/14, 42.9%). Liver involvement was relatively evenly distributed across subtypes, ranging from 14.3% in basal-like/triple-negative tumours to approximately one-third of HER2-enriched cases. Brain involvement was rare overall, but the highest relative frequency was observed in HER2-enriched tumours (3/15, 20.0%). When relapse burden was categorized as single-site versus multisite, no significant association with luminal versus non-luminal grouping emerged. Nevertheless, descriptively, multisite disease was more common in HER2-enriched (9/15, 60.0%) and Luminal B/HER2-positive tumours (11/19, 57.9%) than in Luminal B/HER2-negative disease (21/51, 41.2%). Site-specific relapse patterns according to molecular subtype are illustrated in Fig. 2.

### Timing of PET/ceCT-Detected Relapse

Seven patients were annotated as having metastatic disease at diagnosis. In the subgroup of patients with codable distant relapse and both surgery date and PET/ceCT date available, 86 cases were initially identified; one record showed an implausible negative interval and was excluded from time-to-relapse analysis. In the remaining 85 evaluable patients, the median interval from primary surgery to PET/ceCT-detected distant relapse was 818 days, corresponding to 26.9 months (interquartile range: 261–1749 days, i.e. 8.6–57.5 months). A subtype-specific analysis of time-to-relapse was not performed because complete paired temporal data were not consistently available across all molecular subgroups. This wide dispersion suggests substantial heterogeneity in the timing of distant relapse within the study population.

Clinicopathological characteristics and the overall distribution of PET/ceCT-detected distant relapse are summarized in Table 1. Site-specific relapse patterns according to molecular subtype are reported in Table 2. Bone was the most frequent site of distant relapse overall, whereas luminal tumors showed a predominantly bone-oriented pattern and non-luminal tumors displayed relatively more visceral and nodal involvement.

**Table 1:** Clinicopathological characteristics and overall distribution of PET/ceCT-detected distant relapse. *Percentages exceed 100% because patients with multisite relapse contributed to more than one metastatic category

Variable	*n*/*N*	%
Total study population	177/177	100
Available PET/ceCT relapse-site entry	141/177	79.7
Codable distant relapse on PET/ceCT	137/177	77.4
Primary surgery		
Breast-conserving surgery	51/148	34.5
Mastectomy	63/148	42.6
Biopsy/diagnostic procedure only	32/148	21.6
Other surgery	2/148	1.4
Pathological T stage		
pT1a	1/135	0.7
pT1b	10/135	7.4
pT1c	42/135	31.1
pT2	58/135	43.0
pT3	8/135	5.9
pT4	15/135	11.1
pTx	1/135	0.7
Pathological nodal status		
pN0	41/145	28.3
pN+	94/145	64.8
pNx	10/145	6.9
Molecular subtype		
Luminal A	44/166	26.5
Luminal B/HER2-negative	66/166	39.8
Luminal B/HER2-positive	22/166	13.3
HER2-enriched	19/166	11.4
Basal-like / triple-negative	15/166	9.0
Histology		
Ductal carcinoma	135/161	83.9
Lobular carcinoma	14/161	8.7
Mixed ductal-lobular carcinoma	4/161	2.5
Mucinous carcinoma	4/161	2.5
Other histologies	4/161	2.5
PET/ceCT-detected distant relapse sites*		
Bone	81/137	59.1
Lung	44/137	32.1
Distant lymph nodes	41/137	29.9
Liver	40/137	29.2
Brain	8/137	5.8
Pleura	5/137	3.6
Skin/soft tissue	5/137	3.6
Peritoneal/adnexal	2/137	1.5
Adrenal	1/137	0.7
Other distant sites	1/137	0.7
Relapse distribution		
Single-site distant relapse	70/137	51.1
Multisite distant relapse	67/137	48.9

**Table 2:** PET/ceCT-detected distant relapse pattern according to molecular subtype. Luminal vs. non-luminal tumors: bone involvement was significantly more frequent in luminal disease (65/101, 64.4%) than in non-luminal disease (11/29, 37.9%; *p* = 0.018). Brain metastases were rare overall, but relatively more frequent in HER2-enriched tumors

Molecular subtype	*n*	Bone *n* (%)	Lung *n* (%)	Distant nodes *n* (%)	Liver *n* (%)	Brain *n* (%)	Multisite disease *n* (%)
Luminal A	31	23 (74.2)	8 (25.8)	8 (25.8)	8 (25.8)	1 (3.2)	13 (41.9)
Luminal B/HER2-negative	51	34 (66.7)	16 (31.4)	14 (27.5)	17 (33.3)	2 (3.9)	21 (41.2)
Luminal B/HER2-positive	19	8 (42.1)	7 (36.8)	8 (42.1)	6 (31.6)	1 (5.3)	11 (57.9)
HER2-enriched	15	5 (33.3)	6 (40.0)	6 (40.0)	5 (33.3)	3 (20.0)	9 (60.0)
Basal-like / triple-negative	14	6 (42.9)	6 (42.9)	6 (42.9)	2 (14.3)	0 (0)	7 (50.0)

## Discussion

These results should first be interpreted within the established role of ^18F-FDG PET/CT in the setting of suspected recurrent breast cancer [1, 8, 9]. Previous studies have shown that PET/CT provides clinically relevant information beyond conventional imaging, with meaningful consequences for both management and prognostic stratification. In the study by Cochet et al., PET/CT showed higher negative and positive predictive values than conventional imaging (86% vs. 54% and 95% vs. 70%, respectively), with a high or medium management impact in 57% of patients overall and independent prognostic value for survival [10]. Similarly, the prospective study by Vogsen et al. confirmed high diagnostic accuracy for distant metastases in women with clinically suspected recurrent breast cancer, with sensitivity 1.00, specificity 0.88, and AUC 0.98, while also showing that a negative PET/CT can reliably rule out metastatic disease [11]. Our data do not address diagnostic accuracy per se, because the study was not designed as a head-to-head validation trial and PET and contrast-enhanced CT were interpreted as a single integrated examination [1, 12]. However, they are fully consistent with the idea that PET/ceCT is clinically most informative when used to define the site and extent of relapse rather than merely confirm its presence. The predominance of bone metastases in our cohort is also coherent with the broader breast cancer literature [13]. In a recent population-based analysis, bone was again the most frequent metastatic site, followed by lung, liver, and brain, and the same hierarchy was reported in large clinicopathological series and organotropism-focused reviews [14, 15]. In a recurrence-oriented PET/CT study by Bakhshayeshkaram et al., PET/CT identified additional distant metastases most frequently in the skeletal system, followed by the liver, lungs, and brain, again underscoring the major contribution of whole-body FDG imaging to the detection of osseous disease [16]. Our numbers are directionally similar, although the relative proportion of bone involvement in our series appears even higher. This may reflect case selection, since our cohort specifically includes patients referred for restaging/suspected recurrence, as well as the integrated PET/ceCT interpretation used in daily practice.

The most interesting result of our study is probably the subtype-specific pattern of PET/ceCT-detected distant relapse. Bone involvement was significantly more common in luminal than in non-luminal tumors, and within the individual subgroup analysis the highest relative bone frequency was observed in Luminal A and Luminal B/HER2-negative disease. By contrast, HER2-enriched and basal-like/triple-negative tumors showed relatively more lung, nodal, liver, and brain involvement, as well as more multisite dissemination. This aligns well with contemporary organotropism literature, according to which HR-positive/HER2-negative tumors are more often associated with bone-only or bone-dominant spread, whereas HER2-positive and triple-negative subtypes more frequently develop visceral and brain metastases [14, 15, 17]. Thus, our imaging findings do not simply mirror disease burden; they are biologically plausible and consistent with known subtype-dependent metastatic behavior. The biological relevance of this observation is strengthened by prior evidence that FDG uptake itself is associated with tumor phenotype. Several studies have shown that more aggressive breast cancer phenotypes display higher glucose metabolism, whereas luminal A disease tends to show lower metabolic activity. Şimşek et al. reported the lowest primary-tumor SUVmax values in luminal A tumors and the highest values in HER2-positive disease, with significant associations between FDG uptake, receptor status, grade, proliferation, and outcome [18]. Likewise, Qu et al. showed that SUVmax, metabolic tumor volume, and total lesion glycolysis correlate with adverse clinicopathological factors and poorer prognosis [19]. This is relevant for the interpretation of our results. Although luminal tumors are generally considered less FDG-avid, our data indicate that when recurrence is detected on integrated PET/ceCT, it often manifests as bone-dominant disease. Conversely, more metabolically aggressive non-luminal tumors are more likely to present with visceral or multisite relapse. In other words, the relapse phenotype seen on PET/ceCT probably reflects both organotropism and metabolic aggressiveness. Another point that deserves emphasis is the clinical relevance of PET findings even in luminal disease. Urso et al. demonstrated that in recurrent luminal breast cancer, PET/CT positivity was associated with significantly shorter overall survival and further stratified prognosis independently of CA 15.3 levels [4]. This is important because luminal tumors are often perceived as less informative on ^18F-FDG PET/CT imaging. Our data suggest a complementary message: even in a biologically less avid subgroup, integrated PET/ceCT can reveal a distinct and clinically meaningful relapse phenotype, predominantly bone-oriented, which may still carry important implications for staging and treatment planning. Moreover, the work by Corso et al. showed that increasing CA 15 − 3 and CEA levels are significantly associated with distant metastases detected on ^18F-FDG PET/CT, with a particularly strong association between CA 15 − 3 and bone/liver metastases [20]. Although non-luminal breast cancer subtypes are generally associated with higher ^18F-FDG avidity than luminal tumors, quantitative metabolic comparisons were beyond the scope of the present study, which was specifically designed to investigate site-specific relapse patterns on integrated PET/ceCT. Finally, because tumor markers were not systematically analyzed in our database, these data should be interpreted cautiously; nevertheless, the available literature supports the clinical plausibility of the distribution observed in our cohort and helps frame the role of PET/ceCT in patients referred for suspicion of relapse.

Our findings must also be read in light of an important methodological distinction: this study concerns PET/ceCT, not the isolated PET component alone. This matters especially for liver and lung disease. In routine practice, the hybrid examination benefits from the combination of metabolic whole-body assessment and diagnostic anatomical characterization, and this likely contributed to the identification of multisite and visceral relapse patterns in our cohort. For this reason, the present study should not be interpreted as demonstrating where PET “performs best” in a strict diagnostic accuracy sense. Rather, it describes how integrated PET/ceCT depicts distant relapse in real-world breast cancer restaging. This is a more modest but also more defensible claim.

The present data may also be useful in the context of the evolving PET landscape in breast cancer. Novel tracers are beginning to show subtype-specific advantages over ^18F-FDG in selected settings. For example, in HER2-positive disease, an affibody-based HER2 tracer enabled depiction of more bone, liver, and nodal metastases than ^18F-FDG, whereas lung performance was more similar [21]. These developments are relevant because they suggest that the limitations of ^18F-FDG may not be uniform across biological subgroups or metastatic sites. However, ^18F-FDG PET/CT remains by far the most widely available whole-body oncologic PET technique, and therefore real-world studies such as ours still provide useful information on the imaging phenotype of recurrent disease in everyday clinical practice.

This study has several limitations. First, its retrospective design exposes it to selection bias, and some clinicopathological variables were incomplete. Second, the “site of relapse” field required manual harmonization from free-text entries, which may have introduced some degree of classification bias, especially for uncommon or mixed metastatic patterns. Third, the study was patient-based and descriptive: no lesion-by-lesion histopathological validation was available, and no uniform histopathological confirmation of all sites was obtained. However, potentially equivocal findings and common benign pitfalls, such as fractures or degenerative changes, were interpreted on the basis of integrated PET/ceCT assessment, taking into account lesion morphology, anatomical distribution, and subsequent imaging follow-up when available. Fourth, because PET and contrast-enhanced CT were interpreted together, the present analysis cannot disentangle the specific contribution of metabolic versus anatomical information. Fifth, no standardized lesion-based quantitative comparison of metabolic parameters across molecular subtypes was performed, as the study was specifically designed to investigate site-specific relapse patterns rather than metabolic intensity. Finally, some subtype-specific findings—particularly brain involvement in HER2-enriched disease—were based on small numbers and should therefore be interpreted cautiously. In addition, timing analyses were limited by the incomplete availability of paired surgery and PET/ceCT dates, which prevented a robust subtype-specific evaluation of time-to-relapse. Detailed information on prior systemic treatments, including chemotherapy, endocrine therapy, and HER2-targeted therapy, was not systematically available in the curated database and therefore could not be analyzed as a determinant of relapse timing or site-specific metastatic distribution. Nevertheless, the study also has strengths: it reflects routine clinical practice, uses an integrated whole-body technique, and focuses on a question that is often overlooked in recurrence imaging studies, namely the site-specific phenotype of relapse rather than simple test positivity.

## Conclusions

Our data indicate that distant relapse detected on ^18F-FDG PET/contrast-enhanced CT follows non-random, biologically meaningful patterns in breast cancer. Bone involvement predominates overall and is particularly characteristic of luminal disease, whereas non-luminal tumors display relatively more visceral and multisite relapse. These findings support the use of integrated PET/ceCT as a clinically informative whole-body restaging tool and suggest that the interpretation of relapse imaging should increasingly incorporate molecular subtype and expected metastatic phenotype.

Clinical trial number: Not applicable.

## Data Availability

The datasets generated and/or analyzed during the current study are not publicly available due to institutional and privacy restrictions but are available from the corresponding author on reasonable request.

## Declarations

### Preprint Sharing

This manuscript has not been published or posted as a preprint.
